# Use of rehabilitation pathways in women with breast cancer in the first 12 months of the disease: a retrospective study

**DOI:** 10.1186/s12885-021-07927-0

**Published:** 2021-03-24

**Authors:** Chiara Falcicchio, Domenico Di Lallo, Alessandra Fabi, Alessandro Bonucci, Maria Perrone, Andrea Pace, Ambra Corti, Luca Giacomelli, Patrizia Pugliese

**Affiliations:** 1 Psychology Unit, IRCCS Istituto Nazionale Tumori Regina Elena Roma, Rome, Italy; 2 Direzione Regionale Salute ed Integrazione Socio-Sanitaria, Regione Lazio, Italy; 3 Medical Oncology 1 Unit, IRCCS Istituto Nazionale Tumori Regina Elena Roma, Rome, Italy; 4 Neuroncology Unit, IRCCS Istituto Nazionale Tumori Regina Elena Roma, Rome, Italy; 5 Polistudium srl, Milan, Italy

**Keywords:** Breast cancer, Cognitive, Inpatient, Multidisciplinary, Outpatient, Psychosocial, Rehabilitation

## Abstract

**Background:**

Breast cancer (BC) presents important physical and psychological challenges that should be appropriately addressed through continuous, integrated and individualized rehabilitation programs after treatment. In this study, we aimed to collect more information on the rehabilitation patterns and utilization of healthcare services by women with BC.

**Methods:**

We retrospectively analyzed data from two archives of the Lazio Regional Health System Database to assess rehabilitation patterns in women diagnosed with BC in the Lazio region (Italy) in 2008.

**Results:**

A total of 5538 women diagnosed with BC were considered in the present study. Most patients (81.7%) received outpatient rehabilitative care, consisting mainly of pathology-related interventions and, more rarely, disability-related interventions (mainly motor rehabilitation and rarely cognitive or psychological therapy). Few patients followed an inpatient (1.3%) or an intensive outpatient rehabilitation program (1.0%).

**Conclusion:**

Most patients do not receive adequate rehabilitation care during the first year after diagnosis. More information and better rehabilitation services should be provided to help patients with BC access rehabilitation programs. The study also suggests the importance of psychosocial and cognitive interventions, which is a major unmet need in women with BC.

## Background

Breast cancer (BC) is one of the most common forms of cancer among women and the second-leading cause of cancer-related deaths worldwide [1]. In Italy, it represents the most frequent malignancy in the female population, with approximately 37,000 new cases each year and accounts for 11,000 deaths annually [2].

The recent advances in diagnosis and treatment have considerably improved the survival time and quality of life (QoL) of women with BC [3]. However, women still suffer important physical and psychological challenges that profoundly impact their wellbeing and everyday life [4]. Some of the most frequent complaints reported by women with BC are pain [5], lymphoedema [6], fatigue [7], cognitive issues [8], depression and anxiety, reduced social contacts and challenges in resuming everyday activities and roles after treatment [9–11]. Moreover, younger women reported more unique needs related with gynecological and reproductive consequences, as the issues of fertility and early-menopause and higher levels of distress with their body image, romantic relationships, sexual problems, childcare and career management [12–14]. These unique issues lead to a greater need for information and support care.

Rehabilitation interventions are recognized as an important aspect of care for women with BC. The World Health Organization (WHO) has defined rehabilitation as “the use of all means aimed at reducing the impact of disabling and handicapping conditions and at enabling people with disabilities to achieve optimal social integration”. Based on this bio-psycho-social model, cancer rehabilitation requires a multidisciplinary approach, including medical, psychological and physiotherapeutic treatment, as well as occupational therapy and functional therapy, depending on the specific needs of the patient. The timely delivery of these services, provided both through inpatient or outpatient programs, is crucial to support women after surgery and throughout the post-treatment survivorship stages [15]. These rehabilitation pathways present peculiar challenges related to BC. First of all, they should respond to different needs depending on the patient’s age/stage of the disease (from diagnosis to advanced stages), they should be integrated with ongoing oncological treatments and adapt to different settings, and they should be tailored to the characteristics of the patient (personal history, age, life cycle) and the disease [16, 17]. Useful tools that can be used to support and assess patients during the rehabilitation phases include physician-based and patient-based assessment scales, to clearly identify patient’s needs. The aim of these personalized and flexible paths is to provide an interdisciplinary rehabilitative intervention to patients, reducing or reverting the physical disability, improving functional and cognitive deficit, managing psychological and social issues and optimizing the overall quality of life.

Multiple studies have investigated the effects of different rehabilitation interventions in women who are treated for BC, reporting positive results [18]. According to a recently published systematic review, motor interventions, yoga, complementary and alternative medicine, lymphedema treatment and psychosocial support are the most investigated approaches to rehabilitation in this group of patients [18, 19]. Some of the most effective interventions are exercise programs based on physical activity, which improves shoulder mobility and reduce lymphedema, pain and fatigue, and yoga, which helps women not only on physical terms but also in reducing anxiety, depression and fatigue [20]. The implementation of psychosocial interventions, such as cognitive behavioral therapy, has also reported positive effects on the QoL of women, anxiety, depression, mood disturbances, self-esteem and body image [18].

Despite the availability of multiple studies on rehabilitation interventions, no clear indication exists on which is the best strategy for women with BC. Some complicating factors derive from the heterogeneity of women’s needs and the need of implementing an individualized approach. Moreover, each patient presents a complex mix of mental, psychological, social, emotional, spiritual and physical symptoms caused by the illness and its treatment. All these physical and social psychological factors influence each other according to a cause-effect relationship [17]. Despite the multifaceted aspects of this situation, most studies up to now have investigated only the effects of very specific interventions on detailed and limited outcomes, therefore lacking a more comprehensive evaluation of the complexity of patients’ needs [18].

Furthermore, literature data on the utilization of these services is rather scarce. The few available studies have reported the existence of numerous barriers, which arise from human, logistic and financial sources, which may restrict access to rehabilitative care for women with BC [21, 22].

In order to improve the information on the utilization of rehabilitation services by women with BC, we conducted a retrospective analysis to assess the pattern of rehabilitation care and utilization of public health services by women diagnosed with BC in the Lazio region (Italy) in 2008. This study was part of the research project, Health Organization of Cancer Units for Rehabilitation Activities (HO CURA), supported by the Italian National Ministry of Health 2007–2010.

## Methods

### Patients and methods

In this retrospective cohort study, we collected data from two main sources: 1) the Lazio Regional Health System Database (RAD) and 2) the rehabilitation pathways databases for inpatient (*Sistema Informativo dei ricoveri ospedalieri in Riabilitazione post-acuzie* [RAD-R]) and outpatient interventions (*Sistema Informativo per l’Assistenza Riabilitativa* [SIAR] and *Sistema Informativo per l’Assistenza Specialistica Ambulatoriale* [SIAS]). The RAD collects and stores fully integrated real-world patient-level data on the utilization of health services (including hospital admission, diagnosis and procedures, medical and rehabilitation data). The database includes data from patients admitted to one of the hospitals located in the Lazio region, covering a population of more than 5,000,000 residents. The other databases contain data on rehabilitation programs, categorized as inpatients rehabilitation after hospital discharge for acute disease (RAD-R), intensive rehabilitation pathways provided outside the hospital setting (SIAR) and rehabilitation services provided in the outpatient setting (SIAS).

The RAD-R database, which is an extension of hospital information systems, is aimed at monitoring admissions in case of post-acute intensive rehabilitation at authorized facilities in the Lazio region. RAD-R describes the complexity and specificity of the patient who accesses an intensive post-acute rehabilitation program and reports reasons for access, care commitment, person’s functional dependence and any rehabilitation process following discharge.

The SIAR database is aimed at monitoring extensive rehabilitation activities provided by rehabilitation facilities to describe an overall picture of the rehabilitation projects. This includes people who are in an early post-acute phase and require interventions aimed at ensuring further functional recovery in a defined time, and those suffering from stabilized outcomes of psycho-physical pathologies who need interventions aimed at maintaining any residual functional capacity or containing deterioration.

SIAS database represents the official information source on regional outpatient specialist services; the database guarantees a single and homogeneous detection and description of data. It is essential for the production and dissemination of all the processing aimed at monitoring the activity, financing the facilities and for health planning. For the analysis of SIAS data, according to the protocol, services provided were characterized as related to the needs of the disease (set “Disease”) or linked to a disability (set “Rehabilitation”).

Data from these databases were combined to identify patients diagnosed with BC (ICD-9 code 174) who were admitted to one of the hospitals in the Lazio region and used a rehabilitation service within 12 months after being discharged. Patients were included if they were diagnosed between 1 January 2008 and 31 December 2008, were aged ≥18 years at the time of the first discharge (index date) and received rehabilitation interventions after the index date. We excluded patients with previous hospital admissions for BC (ICD-9 code 174) from the study in the 2 years before 1 January 2008.

### Variables analyzed

Patterns of rehabilitation care were analyzed during a follow-up of 12 months after patients were enrolled in the study, defined as the day of first hospital discharge for BC.

For each patient, we collected data on the rehabilitation services used in the inpatient and/or outpatient settings, including number of rehabilitative interventions delivered between 2008 and 2009, clinical records, length of treatment and time from index date to start of rehabilitation. Data on the inpatient rehabilitation programs included the number of concurrent deficits at admission and the Barthel Index (BI). This score, which ranges from 0 to 100, measures the ability of the patient to perform daily activities of life, with lower scores indicating increasing disability and higher scores indicating greater independence. In the outpatient setting, we collected data on the utilization of neuro-motor rehabilitation programs, and number and type of interventions provided.

### Statistical analysis

Descriptive statistics were used to summarize relevant study information. Categorical variables were reported as frequencies and percentages, while continuous variables were summarized by mean values or median and range, as appropriate.

## Results

During the study period, we identified 11,656 discharges after an initial or secondary diagnosis of BC (ICD-9 code of 174) (Table 1), for a total of 7804 women included in the Regional Health System database of the Lazio region. We selected 5538 women who had no hospital admission for BC in the previous 2 years and were therefore considered as initially diagnosed with BC. For reference, the overall population of the Lazio region in 2008 was of 5,600,000 inhabitants.

**Table 1:** Diagnosis at hospital discharge according to ICD-9 CM code

Main diagnosis	n	%
174.9 – Breast (female) unspecified	2412	43.55
174.4 – Upper–outer quadrant	1087	19.63
174.8 – Other specified sites of the female breast	309	5.58
174.2 – Upper–inner quadrant	258	4.66
174.5 – Lower–outer quadrant	161	2.97
174.1 – Central portion	152	2.74
174.3 – Lower–inner quadrant	147	2.65
174.0 – Nipple and areola	112	2.28
174.6 –Axillary tail	24	0.43
V58.1 –Antineoplastic chemotherapy and immunotherapy	432	7.80
V58.0 – Radiotherapy encounter	26	0.47
Other diagnosis	418	4.55

In total, 90 patients (1.6%) died during the first hospital admission and were excluded from the study; the remaining 5448 women (mean age: 61.2 [47.2–75.2] years) were discharged alive from the hospital and received medical and/or rehabilitation care after the index date. Moreover, during the 12 months after discharge for an acute disease a diagnosis of BC, 70 (1.3%) women followed an inpatient rehabilitation program, 54 (1.0%) were included in an intensive outpatient rehabilitation program and 4452 (81.7%) required specific outpatient rehabilitative care. Data on the rehabilitation care provided to patients in the 12 months after discharge are summarized in Table 2.

**Table 2:** Rehabilitation care provided in the 12 months after discharge

Rehabilitation care	Patients, n (%)	BI score at the beginning of rehabilitation; median (IQR)	Time between diagnosis and start of rehabilitation (% of patients)	Length of rehabilitation; median (IQR)
Inpatients rehabilitation:				
Ordinary hospitalization Standard	70 (1.3)		Within 30 days: 78%	41 days (22–58)
Day hospital:		52 (31–77)	Within 30 days: 50%	
• Locomotion deficit	31 (44.1)			
• Urinary deficit	31 (44.1)			
• Manipulation deficit	26 (36.9)			
Intensive outpatient rehabilitation:	54 (1.0)	90 (72–96)	Within 12 months	53 (49–79)
• Neuro-motor rehabilitation	45 (82.6)			
• Cardiologic rehabilitation	9 (15.9)			
• Psychologic rehabilitation	6 (8.7)			
Outpatients rehabilitation^*^:				
Disease:	3604 (81)	NA	Within 6 months: 47.9%	39 days
• Cure	3199 (71.9)		Within 6 months: 42.5%	18 days
• After-care	654 (14.7)			
• Follow-up	591 (13.3)			
Rehabilitation:	22 (0.5)			
• Improvement of motor functioning/behaviors	18 (81.5)			
• Counseling, support or cognitive behavioral therapy	2 (5.6)			
• Pain reduction and prevention	1 (5.0)			

BI: Barthel index; IQR: Interquartile range; NA: Not available

*Disease: interventions focused on disease-related needs; Rehabilitation: disability-related interventions

### Inpatient rehabilitation program – RAD-R database

In total, 70 patients (1.3%; mean age 68 ± 15 years old) were admitted to an inpatient rehabilitation program after hospital discharge, for a total of 84 admissions (59 standard admissions and 25 day-hospital admissions). Regarding standard admissions, in 78% of the cases the first admission occurred within 30 days from discharge, and the mean duration of hospital stay was 41 days (interquartile range [IQR]: 22–58) (Table 2). On the other hand, 50% of day hospital admissions occurred within 30 days from discharge.

The median BI score measured before the start of rehabilitation was 52 (IQR:31–77) (Table 2), indicative of a low functioning dependence in daily living activities. The most common deficits reported by patients were related to locomotion (44.1%), urinary system (44.1%), and manipulation (36.9%). Notably, 91.7% of all the patients admitted presented at least one deficit, 50% reported at least two deficits and 25% reported at least four.

After discharge at home (occurred in 73.8% of women), 41.1% of the patients did not follow an additional rehabilitation path, 13.0% followed a standard inpatient rehabilitation program and 27.3% an outpatient program.

### Intensive outpatient rehabilitation plan – SIAR database

A total of 54 patients (1%; mean age 61 ± 14 years old) were addressed to a personalized outpatient program with intensive rehabilitation plan or maintenance plan. Overall, 69 rehabilitation programs were delivered, including 28 intensive programs (40.6%) and 41 maintenance programs (59.4%), classified as requiring low (17.4%), medium (53.6%) or high interventions (29%). Most of the patients were referred to the program by their general practitioners (91.3%), while in the remaining 5.8% of the cases the rehabilitation program was prescribed by a specialist.

The median BI score measured before the start of rehabilitation was 90 (IQR: 72–96) (Table 2), indicative of a low dependence in daily life functioning activities. In our population, 82.6% of the patients followed a rehabilitation program focused on neuro-motor rehabilitation, 15.9% a cardiologic rehabilitation, and the remaining 8.7% focused on psychological rehabilitation (Table 3). Notably, 66.7% of the patients followed these programs in an outpatient setting, while 20.3% of the patients required a specific high-care assistance at their domicile.

**Table 3:** Type of interventions delivered in intensive rehabilitation programs

Intervention	n	%
Neuromotor rehabilitation	57	82.6
Cardiologic rehabilitation	11	15.9
Psychologic rehabilitation	6	8.7
Educational intervention	5	7.2
Nurse intervention	5	7.2
Occupational therapy	2	2.9
Logopedic rehabilitation	1	1.4

The median number of effective days in which the rehabilitation program was delivered was 53 (IQR: 49–79) (Table 2). After the end of the rehabilitation plan, most of the women continued a different rehabilitation program (47.8%), while no additional program was proposed to 39.1% of the patients.

### Outpatient rehabilitation services – SIAS database

After hospital discharge, a total of 4452 (81.7%) women were followed through specialized outpatient rehabilitation programs and received overall 187,680 rehabilitation interventions and 102,086 prescriptions during the study period. In the majority of the cases (81%), the rehabilitation interventions focused on disease-related needs (set “disease”), in 0.5% of the cases consisted of disability-related interventions (set “rehabilitation”) and 18.6% of the patients received both disease and disability-related support (Table 2).

Overall, 172,482 disease-related interventions were provided during the study, divided in cure (71.9%), after-care (14.7%) and follow-up (13.3%). A total of 72% of the interventions consisted in medical/surgical services to treat the neoplastic disease or its collateral effects, 14.7% were related to the treatment of collateral effect or management of medical devices (mainly delivered by nurses) and 13.3% consisted in medical-surgical monitoring of patients’ status (Table 4). These interventions usually started 46 (IQR: 23–103) days after the index case and were provided within 6 months from discharge in 43% of the cases (Table 2). The mean number of effective days in which these interventions were provided was 39, with 86.9% of the women continuing the program for at least 6 months (Table 2).

**Table 4:** Type of interventions delivered in outpatient rehabilitation programs

Interventions	n	%
***Disease-related interventions***		
After care	25,374	14.7
Cure	124,146	71.9
Follow-up	22,962	13.3
***Disability-related intervention***		
Instrumental evaluation of function	1148	7.5
Exercises/occupational therapy/physio-kinesitherapy	12,379	81.4
Pain therapy	762	5.0
Cognitive evaluation/therapy	57	0.3
Support/psychologic therapy	852	5.6

Regarding disability-related interventions, which amounted to 15.198 in total, 81.5% were related to improvement of motor functioning and behaviors; 5.6% consisted in counselling, support or cognitive behavioral therapy; 5.0% were directed to pain reduction and prevention through physical means (percutaneous electrotherapy, epidural electrostimulation or massage therapy) and 0.4% consisted in the evaluation of cognitive functioning through tests or interviews (Table 4). These interventions started on average 161 (IQR: 75–250) days after the index case and were provided within 6 months from discharge in 42.5% of the cases. The mean number of effective days in which these interventions were provided was 18 days, with 28.8% of the women continuing the program for at least 6 months.

Notably 58% of the disease-related interventions and 46.0% of disability-related interventions were provided in local district services near the hospital in which women were initially admitted, whereas 39.8 and 70.2%, respectively, were provided near women’s domicile.

## Discussion

Women diagnosed and treated for BC present both physical and psychosocial challenges that negatively influence their wellbeing and QoL [4].]. Appropriate rehabilitation programs should be implemented to follow these women from the time after surgery through the survivorship phase, to help them achieve the highest possible independence and the best QoL. These programs should focus mainly on managing physical disability, reducing sequelae and symptoms, and enhancing psychological health and societal reintegration [21]. Given the complexity of patients’ needs, it is essential to implement a multidisciplinary approach to respond to the oncological, functional and psychosocial needs of the patients. Although no clear indication exists in literature on which experts should be part of this multidisciplinary team it seems to be important to include oncology specialists (e.g., breast surgeon, plastic surgeon, pathologist, radiologist, medical oncologist and radiation oncologist), doctors specialized in functional and physical re-conditioning and experts in the psycho-educational aspects of cancer survivorship [22–24].

Although various studies have investigated the efficacy of different motor and cognitive rehabilitation programs for women with BC, only a limited number of studies have focused on the utilization of these services [23–25]. A comprehensive picture and understanding of the availability and use of these services is therefore still lacking, and no clear information exists on the most appropriate duration of rehabilitation services for BC. These evaluations are complicated by the complexity of the disease, the differences in perceived needs and the importance of implementing an individualized multidisciplinary rehabilitative strategy.

In this study, we focused on the utilization of rehabilitation services in women diagnosed with BC in the Lazio region in 2008. For reference, in 2008, there were approximately 50,000 women diagnosed with BC in the Lazio region, thus making this region the second highest with regards to incidence of BC in Italy [2].

The results of our retrospective study show that the vast majority of BC patients do not have access to inpatients and personalized rehabilitation services in the first year after the first discharge. Only 1.3% of the patients with a moderate disability score (median BI = 50), accessed an inpatient rehabilitation program, whereas an even smaller percentage (1.0%) received a personalized intensive outpatients rehabilitation plan. Most of the patients (81.7%) received specialist care in the outpatient setting, consisting mainly in pathology-related interventions and only in a limited number of cases in disability-related interventions. Of note, disability-related interventions were delivered long after hospital discharge (median: 161 days) and usually later compared with disease-related services (median: 45 days). Moreover, our findings suggest that women with BC received fragmented rehabilitation services, in particular in the outpatient setting. It is important to note that literature data suggest that outpatient rehabilitation programs are as beneficial to patients as inpatients programs, but with an important cost saving for the National Health System, thus suggesting the need for a more consistent implementation of rehabilitation services in this setting [26].

One of the main services provided to women in the outpatient setting, accounting for 81.5% of all the disability-related interventions, was motor rehabilitation, consisting in exercise, occupational therapy and physio-kinesiotherapy. Exercise and physical activity interventions are the most studied type of rehabilitation; according to literature data, they are indeed feasible, safe and effective in improving women’s physical outcomes, and have also reported predominantly positive effects on fatigue and QoL [27, 28]. Conversely, only a limited percentage of women received cognitive therapy (0.3%) or psychological support (5.61%). Literature data suggest that while physical functioning improves after surgery in women treated for BC, the cognitive, emotional and social functioning of QoL do not significantly improve after treatment [29, 30]. These observations underline the importance of implementing psychosocial rehabilitation programs to help women reduce their depressive symptoms, maintain close relationship and help them to recover breast-specific functions, such as body image and sexual life [9].

The limited use of inpatients and especially personalized intensive outpatient’s rehabilitation plans observed in our retrospective analysis suggest the lack of rehabilitative offer and the existence of barriers to patients’ access. Although only few studies have specifically evaluated the barriers to rehabilitation in women with BC [21, 22], literature data in the cancer population suggest that lack of awareness and lack of physician referral are the main causes that restrict the use of rehabilitation services [31]. In the case of BC, we also believe that the scant attention payed by Institutions to oncology rehabilitation, the limited knowledge on BC peculiarities and challenges, and the poor interactions between hospitals and local health services may represent a significant barrier to the development of new programs, as shown by the exclusion of oncology rehabilitation plans from the minimal level of care (Livelli Essenziali di Assistenza [LEA]) in Italy [32]. We believe that other important barriers that may hamper the development of BC rehabilitation programs in Italy include the scant education and information on multidisciplinary strategies, the lack of a standardized approach to assess patient’s specific rehabilitation needs (e.g., through patient- or physician-based tools) and the insufficient information provided by evidence-based studies and randomized trials on which are the most effective interventions [21, 22].

Increasing the level of information and understanding of oncological rehabilitative services is an important step to overcome the existing barriers and to promote a more equal and comprehensive access of rehabilitation interventions in women with BC [22].

### Strengths and limitations

We acknowledge some limitations of this study, including the retrospective design and the fact that the source of this analysis are administrative data, which lack compete clinical information. On the other hand, by using the archives of the Lazio Regional Health System, we could evaluate the utilization of rehabilitation services in a large cohort of patients, and we had access to a comprehensive database of rehabilitative pathways, from post-acute hospital admissions up to outpatient treatments.

In addition, the long interval from data collection and our analysis could be considered a possible limitation of the study; however, no relevant changes have been made over the last 10 years in the regional health system in terms of availability of public rehabilitation services. Consequently, presented data are still representative of the current clinical practice.

## Conclusion

Our retrospective analysis on the utilization of rehabilitation services in a large cohort of women with BC clearly shows that the majority of women do not receive adequate rehabilitation care during the first year after diagnosis. These results underline the need of improved availability of rehabilitation services and of further information to help BC patients access rehabilitation programs; the study also suggests that more focus should be put on psychosocial and cognitive interventions, which is a major unmet need in women with BC.

## Data Availability

The datasets used and/or analysed during the current study are available from the corresponding author on reasonable request.

## Abbreviations

BC: Breast cancer
BI: Barthel Index
HO CURA: Health Organization of Cancer Units for Rehabilitation Activities
IQR: interquartile range
LEA: Livelli Essenziali di Assistenza
QoL: Quality of life
RAD: Lazio Regional Health System Database
RAD-R: Sistema Informativo dei ricoveri ospedalieri in Riabilitazione post-acuzie
SIAR: Sistema Informativo per l’Assistenza Riabilitativa
SIAS: Sistema Informativo per l’Assistenza Specialistica Ambulatoriale
